# The Dental Exhibit at the Centennial

**Published:** 1876-11

**Authors:** 


### EDITORIAL, ETC.

*The Dental Exhibit at the Centennial.*—In the *Dental Advertiser* we find the following description of the dental display at the Centennial:

"The great Industrial Exhibition, now open at Philadelphia, affords a wonderful display of the proficiency of different nations in the arts and sciences. It is bewildering from its vastness, and the visitor becomes weary of sight-seeing, and returns to his home, often without a passing glance at even half the wonders and beauties waiting for his inspection. The exhibition there made of the art of dentistry, affords to those interested in it, an instructive token of its progress and present status, and it is scarcely credible that so short a time as fifty years has been sufficient to raise it from being in great part the business of vagabonds and itinerants, to the proud position it now occupies.

A mention of the names of exhibitors and a short description of their displays may not prove uninteresting to those of the profession who find themselves unable to attend the Exhibition in person. It is needless to say that in this branch, more perhaps than in any other, the United States of America overshadows all other nations. The exhibit would be meager indeed if contributions from our country were removed.

The largest and most complete display made by any manufacturer of dental material, is that of Samuel S. White, of Philadelphia, whose exhibition includes, in addition to a full assortment of teeth, gold foil and the usual instruments, several novelties; among which the new S. S. White chair and engine occupy a prominent position, the latter being also shown as driven by a water motor, and by electricity. In a word, there is scarcely anything which can be named as included in a dental outfit which cannot be found in his collections.

Messrs. Codman & Shurtleff, of Boston, Mass., who are large manufacturers both of surgical and dental instruments and appliances, have a truly valuable and fine looking display of their specialties. Their stock of forceps, excavators and pluggers have an excellent finish, and are well worthy of more than a passing glance. They show floss silk holders and foil carriers of their design, struck up from thin steel, very light but sufficiently strong for use ; mouth mirrors, inhalers for nitrous oxide, ether and chloroform, chairs, brackets and spittoons, and a very neat and compact form of operating case. The reputation of this firm stands deservedly high for the excellence of their manufactures. Their exhibit is in charge of an attendant, who is always ready to allow to those who wish, a close examination of the contents of the cases.

H. D. Justi, of Philadelphia, has an elegant case, presenting a full assortment of teeth of his manufacture and the materials of which they are composed. The mode of arrangement of the teeth in the cases is very striking. and displays them to great advantage. Several new moulds are to be noticed among the number, betokening the efforts constantly put forth by this gentleman to excel in his department.

Messrs. Johnson & Lund, of Philadelphia, have two cases in which they show artificial teeth, corundum wheels, gold foil, tooth powder, amalgam,.and dental instruments.

Gold is usually a very attractive metal to any of the human race, whether belonging to the dental profession or not, and it is unusually attractive in appearance in the case which contains samples of the manufactures of Wm. Valleau, Jr., and Geo. J. Pack, of New York City. Gold and silver leaf, and the various preparations of gold which they furnish for dental use are shown, arranged with great taste, and displaying some unique effects. A miniature log cabin made of gold cylinders, and a statuette formed of Pack's pellets, deserve special mention. We are unable to say whether the statuette in question is intended as an effigy of the manufacturer of the material of which it is composed, or of the late G. Washington, Esq. It resembles either in some respects.

Messrs. Charles Abbey & Son, of Philadelphia, exhibit a case of gold foil, and the medals they have obtained at different fairs in which they have taken a part.

D. W. Neal, of Camden, N. J., shows a case of artificial teeth.

Dr. W. G. A. Bonwill, of Philadelphia, has on exhibition his electro-magnetic plugger for filling teeth, an instrument which is attracting considerable notice, and has some very warm advocates. He has previously been awarded the Cresson gold medal, a prize given only for invention or discoveries deemed of especial merit, which has been awarded but six times in twenty-nine years.

Horatio G. Kern, of Philadelphia, exhibits a case containing a full line of forceps and instruments of his manufacture.

Dr. S. Wardle, has on exhibition some very fine specimens of carved block work, which are well worthy of attention, under a sign bearing the inscription, 'Not attempted a hundred years ago.'

Dr. Thomas Wardle, of Philadelphia, presents a case containing a number of models illustrative of the resources of the dentist in the correction of irregularities of the teeth. There are some very fine studies among the cases presented.

Probably the best opportunity of contrasting the past and the present of dental science, is afforded in the case of Dr. John Allen, of New York. An upper and lower set of teeth once worn by General Washington, contributed by the Baltimore College of Dental Surgery, and a lower set also worn by the General, contributed by Mr. Isaac Greenwood, of New York, and a set said to have been worn by Aaron Burr, can there be contrasted with the doctor's latest achievements in continuous gum work. The contrast is very interesting and instructive.

Dr. E. Parmly Brown, of New York, the inventor of the depressed rubber dam, exhibits in addition to that article, three cabinets of his own design, some fine specimens of gold fillings, and some other articles which we believe were invented by the doctor, which are well worthy of a more extended notice than our space will allow.

Dr. Quincy A. Scott, of Pittsburg, Penn., shows his atmospheric disc for retaining plates in the mouth.

Dr. Thomas B. Gunning, of New York, has an assortment of appliances for reducing fractures of the jaw, correcting irregularities, etc.

Dr. Charles A. White, of Philadelphia, has a celluloid apparatus of his own design, flasks, etc., on exhibition.

Dr. Volney Smith, Newark, N. J., exhibits in the English department whole and partial sets of teeth mounted on the Coralline base.

Italy is represented by 'Leopoldo Gramignana, Chirurgica Dentista,' with a small case of artificial dentures.

Dr. Noel Winderling, of Milian, presents a very fine Dental Museum, containing models illustrative of the development of the teeth, the diseases of the teeth and jaws, cleft palate, etc.

The object of the collection is to present at one view, the whole history of the teeth and their adjacent parts, whether in a physiological or pathological state. The material here brought together could scarcely have been found without great labor and persistence, and the case would be a valuable acquisition to a dental school.

In the Buenos Ayres department may be found a case containing artificial teeth, mounted by 'Dr. Rodolfa Newbery, Dentista Americano.'

Dr. F. A. Berghamer, of Vienna, Austria, presents a case similar to the above.

In the Russian department are three small exhibits by different dentists.

All the foregoing exhibits are to be found in the main building, but scattered through the departments belonging to the different nationalities. There are two other cases, which will be found in the women's pavilion, their contents being the handiwork of lady dentists.

Mrs. Dr. F. C. Treadwell, of Philadelphia, has a case containing specimens of gold and other fillings. We understand she has been in practice since 1854.

Annie D. Ramborger, D. D. S., a regular graduate of dentistry, exhibits specimens of gold fillings and gold plate work.

It will be seen from the foregoing resume that the dental exhibit, taken as a whole, is an interesting one, when we take into account the fact the great reluctance to figuring in exhibitions of this sort which exists with many, indeed a majority of the more intelligent among the profession. A fuller representation of Manufactures from foreign parts would have greatly enhanced its value, affording as it would, opportunity for comparison with our home productions. As it is, however, not a single foreign manufacturer of dental goods has ventured to put his wares in competition with those furnished by this country."

